# N7-methylguanosine-related lncRNAs: Distinction between hot and cold tumors and construction of predictive models in colon adenocarcinoma

**DOI:** 10.3389/fonc.2022.951452

**Published:** 2022-09-15

**Authors:** Zhichao Cheng, Jiaqi Wang, Yixin Xu, Tao Jiang, Zhenyu Xue, Shuai Li, Ying Zhao, Hu Song, Jun Song

**Affiliations:** ^1^ The Graduate School, Xuzhou Medical University, Xuzhou, Jiangsu, China; ^2^ Department of General Surgery, The Affiliated Hospital of Xuzhou Medical University, Xuzhou, Jiangsu, China; ^3^ Institute of Digestive Diseases, Xuzhou Medical University, Xuzhou, Jiangsu, China; ^4^ Department of General Surgery, Southwest Hospital, Third Military Medical University, Chongqing, China

**Keywords:** colon adenocarcinoma, m7G, lncRNA, hot tumor, cold tumor, model

## Abstract

Colon adenocarcinoma (COAD) is a prevalent malignant tumor that severely threatens human health across the globe. Immunotherapy is an essential need for patients with COAD. N7-methylguanosine (m7G) has been associated with human diseases, and non-coding RNAs (lncRNAs) regulate various tumor-related biological processes. Nonetheless, the m7G-related lncRNAs involved in COAD regulation are limited. This study aims to construct the clustering features and prognostic model of m7G-related lncRNAs in COAD. First, The Cancer Genome Atlas (TCGA) database was used to identify m7G-related differentially expressed lncRNAs (DELs), based on which COAD cases could be classified into two subtypes. Subsequently, univariate Cox analysis was used to identify 9 prognostic m7G-related lncRNAs. Further, Five candidates were screened by LASSO-Cox regression to develop new models. The patients were divided into high-risk and low-risk groups based on the median risk score. Consequently, the Kaplan-Meier survival curve demonstrated a statistically significant overall survival (OS) between the high- and low-risk groups (P<0.001). Multivariate Cox regression analysis revealed that risk score is an independent prognostic factor in COAD patients (P<0.001). This confirms the clinical applicability of the model. Additionally, we performed Gene Set Enrichment Analysis (GSEA), which uncovered the biological and functional differences between risk subgroups, i.e., enrichment of immune-related diseases in the high-risk group and enrichment of metabolic-related pathways in the low-risk group. In a drug sensitivity analysis, high-risk group were more sensitive to some chemotherapeutics and targeted drugs than low-risk group. Eventually, the stability of the model was confirmed by qRT-PCR. Our study unraveled the features of different immune states of COAD and established a prognostic model, including five m7G-related lncRNAs for COAD patients. These results will bolster clinical treatment and survival prediction of COAD.

## Introduction

Colorectal cancer is a prevalent gastrointestinal cancer with a 3^rd^ leading incidence and a 2^nd^ leading mortality rate as per the 2020 global statistics (1). Colon adenocarcinoma is the most common type of colon cancer (2). Although the overall morbidity and mortality rates of colon cancer have declined in the past two years, this progress is increasingly limited to the elderly population (3). The early diagnosis and prognosis of colon cancer patients are understudied (4). Tumor-lymph node-metastasis (TNM) staging system is a conventional method for predicting colon cancer (5) with limitations. Therefore, individualized evaluations are used in patients with colorectal cancer at an early stage, including molecular and immune characteristics of the tumor. Additional analysis is necessary to improve patient outcomes (6).

N7-methylguanosine (m7G) has been isolated in the 5 ‘ end cap of eukaryotic mRNA and the internal position of tRNA and rRNA (7). In most cases, m7G modification occurs at the 46^th^ position of the variable region, a product of tRNA methyltransferase that can help stabilize tRNA structure (8). Additional studies have shown that this modified form of m7G potentially triggers the occurrence of colon and lung cancers. Methyltransferase-like 1 (METTL1)-mediated m7G regulates the progression and chemosensitivity of colon cancer (9). The expression levels of the tRNAm7G methyltransferase complex component, METTL1, and WD repeat domain 4 (WDR4) were significantly upregulated and threatened the survival of patients. METTL1 promotes the growth and invasion of lung cancer by regulating m7GtRNA modification; however, its specific mechanism warrants additional studies (10).

LncRNAs are non-coding RNAs longer than 200 nucleotides in length (11). Many studies have shown that lncRNAs modulate various tumor-related biological processes (12–14). Studies have shown that abnormal lncRNA expression is a marker for determining tumor prognosis and diagnosis (15). Nevertheless, the available evidence on lncRNAs in m7G modification in colon adenocarcinoma is limited. Herein, we explored relevant characteristics of different immune states of colon adenocarcinoma to establish a reliable prognostic model.

## Materials and method

### Data acquisition

Transcriptomic and clinical data of 398 colon adenocarcinoma tissue samples and 39 normal colon samples were downloaded from the TCGA database, respectively, from the literature and GSEA database (http://www.gsea-msigdb.org/gsea/login.jsp) were obtained for 40 m7G-related genes (**Supplementary Table 1**). Patients with a follow-up period of fewer than 30 days were excluded to minimize errors in the statistical analysis.

### Selection of m7G-related lncRNAs

The lncRNAs of 398 patients with colon adenocarcinoma were screened using Strawberry Perl (version 5.30.0), and m7G-related lncRNAs were identified using the limma R package Pearson correlation coefficient > 0.4 and P< 0.001 as conditions. To screen for differentially expressed lncRNAs (DELs), normal colon tissues and colon adenocarcinoma tissues were filtered based on specific criteria (|log 2 FC| > 1 and FDR< 0.05)

### Consensus clustering and its immune signature

Consensus clustering was performed using the ConsensuClusterPlus R package (16–18). The immune microenvironment of tumor tissue was evaluated using the estimate R package, and we calculated the evaluation scores of immune and stromal cells. The pan-cancer immune cell infiltration files were downloaded from TIMER 2.0 and used to analyze the distribution of the explored immune cells on different clusters with the help of the limma and pheatmap R packages (19, 20). The limma and ggpub R packages were used to perform the differential analysis of the immune microenvironment and immune checkpoints.

### Construction and validation of an m7G-associated lncRNA-related prognostic model

A total of 398 colon adenocarcinoma patients were randomly divided into training and validation groups in a 1:1 ratio. Then, DELs for differential analysis were filtered using univariate Cox regression analysis (P< 0.01); this further defined the potential prognostic related lncRNAs. LASSO-Cox regression analysis generated the best m7G-related lncRNA prognostic risk model for colon adenocarcinoma (21). The risk score was calculated using the following formula:


$$risk\;score=\sum_{i=1}^{n}coef_{i}*expr_{i}$$

Where expr_i_ represents the expression level of each lncRNA, and coef_i_ is the regression coefficient of each lncRNA in this model. Patients in the training group were subdivided into high-risk and low-risk groups as per the median of the training group risk scores. Kaplan-Meier curves were drawn using the survminer R package (22). The log-rank test was used to compare the survival status of the two groups. Subsequently, time-dependent receiver operating characteristic curves were generated using the time ROC R package. The area under the curve (AUC) was calculated for 1-, 3-, and 5-year overall survival. To check the feasibility of this model, the risk score in the validation set was calculated using the same formula as in the training set. Besides, we used a similar validation method as above. Finally, univariate and multivariate Cox regression analyses of clinicopathological factors, including age and stage, were performed to test the independent predictive capacity of the model for the prognosis of patients with colon adenocarcinoma. Subsequently, a nomogram was constructed combining the above factors, and a calibration curve was used to test the suitability of this nomogram.

### Specimen acquisition

The colon adenocarcinoma tissues and adjacent tissues of 24 patients were collected from the Affiliated Hospital of Xuzhou Medical University between 2012 and 2014. These patients did not receive any radiotherapy or chemotherapy before surgery, and the diagnosis was confirmed through postoperative pathological examination. This study was approved by the Ethics Committee of the Affiliated Hospital of Xuzhou Medical University, and the experiments adhered to the Declaration of Helsinki. Each patient provided written informed consent. All excised specimens were immediately stored in liquid nitrogen.

### Quantitative real-time PCR

Total RNA from tissues was extracted and quantified using the TRIzol reagent (Takara, China) following the manufacturer’s instructions. Thereafter, total RNA was reverse transcribed into cDNA using PrimeScrip RT Master Mix (Takara) as per the manufacturer’s protocol. Three replicates were set for each sample, and the 2-ΔΔCT method was used for data analyses. **Supplementary Table 2** shows the primer sequences used for analysis.

### Statistical analysis

Statistical analyses were performed using R 4.1.2 and GraphPad 9.0.0. The Kaplan-Meier method was used to calculate the survival rate of patients with COAD. Moreover, univariate and multivariate regression analyses were performed to evaluate the association of risk scores and clinical characteristics with overall survival. P values of less than 0.05 (P<0.05) were considered statistically significant (*P< 0.05, **P< 0.01 and ***P< 0.001).

## Results

### Screening of differentially expressed m7G-related lncRNAs in COAD

**Figure 1:**
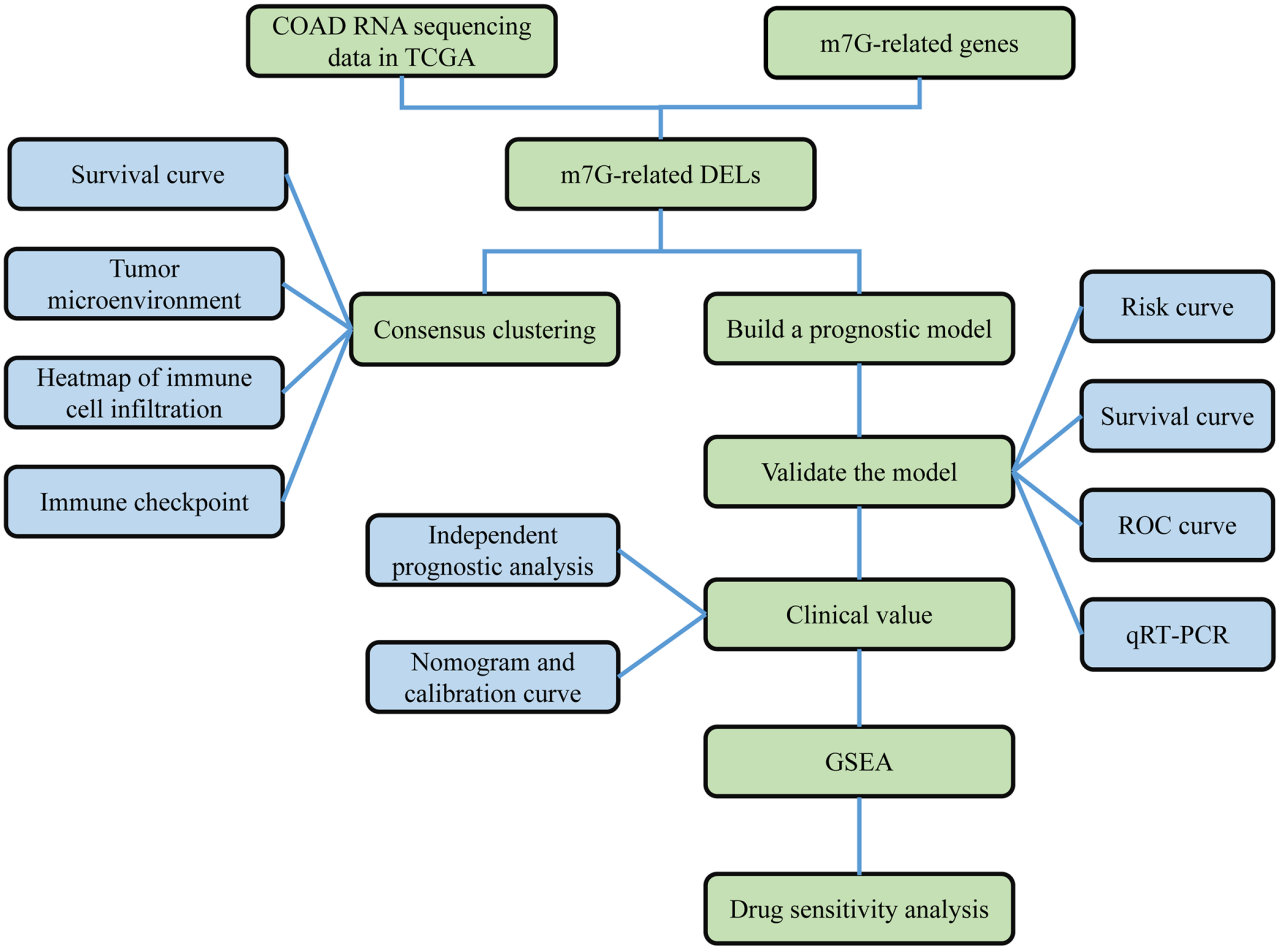
Work flow chart. First, RNA sequencing data of COAD were obtained from the TCGA database, and 40 m7G-related genes were obtained from the GSEA database and published literature. Then, 990 m7G-related lncRNAs were identified based on differential analysis. Next, COAD was classified based on DELs, and survival analysis and immune correlation analysis found differences in survival and immune characteristics of different clusters. In addition, LASSO-COX analysis was applied to screen for prognosis-related lncRNAs. Based on this analysis, a prognostic risk model was constructed. The stability of the model was verified using survival analysis, ROC curve, nomogram, GSEA and qRT-PCR.

**Figure 1** shows our research flow chart. Data on 398 colon adenocarcinoma samples were downloaded from the TCGA database; 14,056 lncRNAs and 19,673 mRNAs were identified. Also, 40 m7G-related genes were obtained from relevant literature (23) and the GSEA database (http://www.gsea-msigdb.org/gsea/login.jsp). Differential analysis of m7G-associated lncRNAs was performed, and 903 DELs (|log2 FC| > 1 and FDR<0.05) were screened, including 807 up-regulated lncRNAs and 96 down-regulated lncRNAs; volcano plots were drawn (**Figure 4A**).

### DELs-based tumor classification

**Figure 2:**
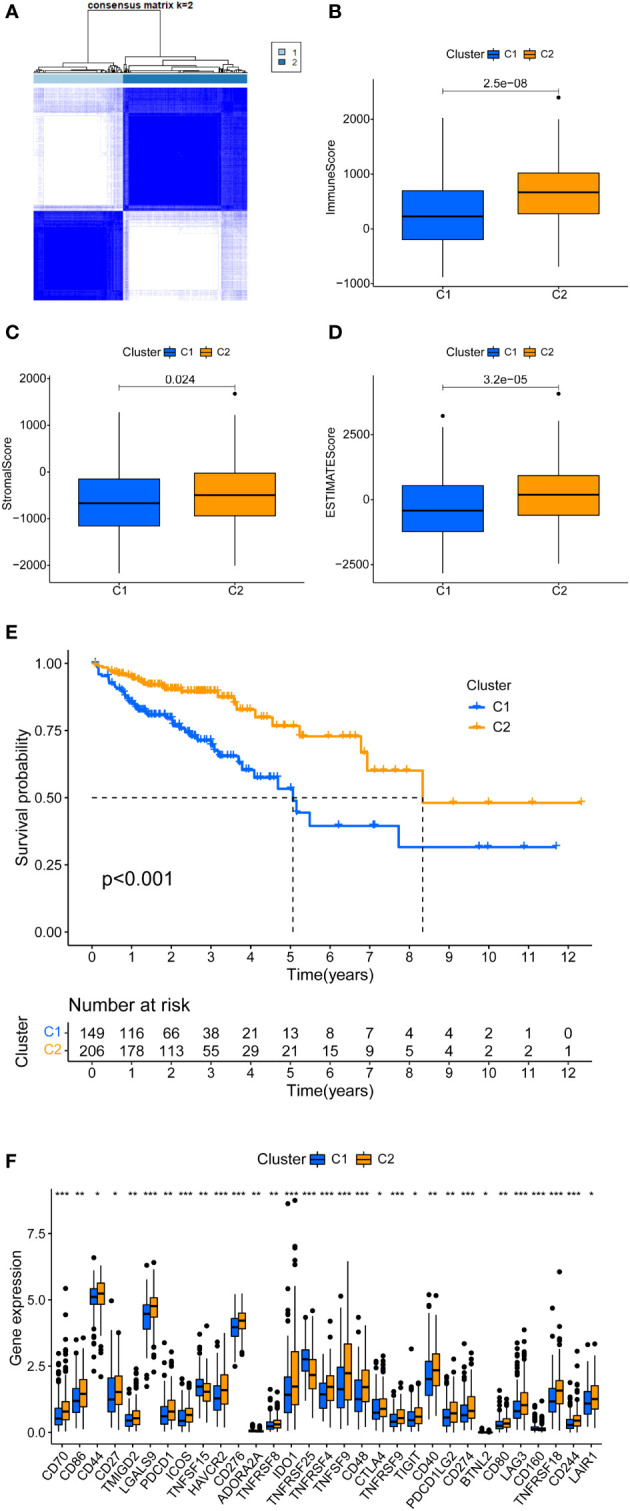
Tumor typing based on m7G-related DEL. **(A)** COAD patients were divided into two clusters. **(B-D)** Significant differences in the tumor microenvironment of Cluster 1 and Cluster 2. **(E) **There is a clear difference in the survival curves of Cluster 1 and Cluster 2 (P < 0.01). **(F)** Immune checkpoint gene expression differences between cluster 1 and cluster 2. *P < 0.05, **P < 0.01 and ***P < 0.001.

Machine learning-based unsupervised consensus was used to cluster 398 COAD patients in the TCGA cohort to explore the relationship between m7G-related DELs and COAD subtypes (16, 18). By increasing the clustering variable (k) from 2 to 9, we found that when k =2, the within-group correlation was highest, whereas the between-group correlation was low (**Figure 2A**). Therefore, all COAD patients were divided into two clusters, i.e., Cluster1 and Cluster2. Kaplan-Meier survival analysis showed that the OS of Cluster1 was significantly shorter than that of Cluster2 (**Figure 2E**). DELs and clinical features between the two clusters were presented in a heatmap, where a significant difference was found in the stage between the two clusters (**Supplementary Figure 1A**). This confirms that COAD patients can be divided into two clusters.

### Immune correlation analysis based on consensus clustering

In clinical practice, a few patients are not sensitive to immunotherapy. Conventional assays include tumor mutational burden, mismatch repair/microsatellite instability, and expression of immune checkpoint genes. We added new detection methods for a more accurate application of immunotherapy. First, the tumor microenvironment scores of COAD patients were calculated, including immune score, stromal score, and ESTIMATE score. **Figures 2B-D** shows the differential analysis of the tumor microenvironment; Cluster2 had higher immune, stromal, and ESTIMAT scores. The immune correlation heatmap showed the distribution of immune cells in different clusters. CD8+ T cells from TIMER, CIBERSORT-ABS, QUANTISEQ, and XCELL had significantly more infiltration in Cluster2 (**Supplementary Table 3**; **Supplementary Figure 1B**) (24). Several immune checkpoints were significantly higher in Cluster2 than Cluster1, specifically PD1, PD-L1, CTL4, LAG3, and TIM3 (**Figure 2F**). Collectively, Cluster2 had a higher tumor microenvironment score, immune cell infiltration, and immune checkpoint expression. Therefore, Cluster2 is a hot tumor, whereas Cluster1 is a cold tumor (25). Notably, hot tumors are sensitive to immunotherapy, unlike cold tumors.

### Development and validation of prognostic model

**Figure 3:**
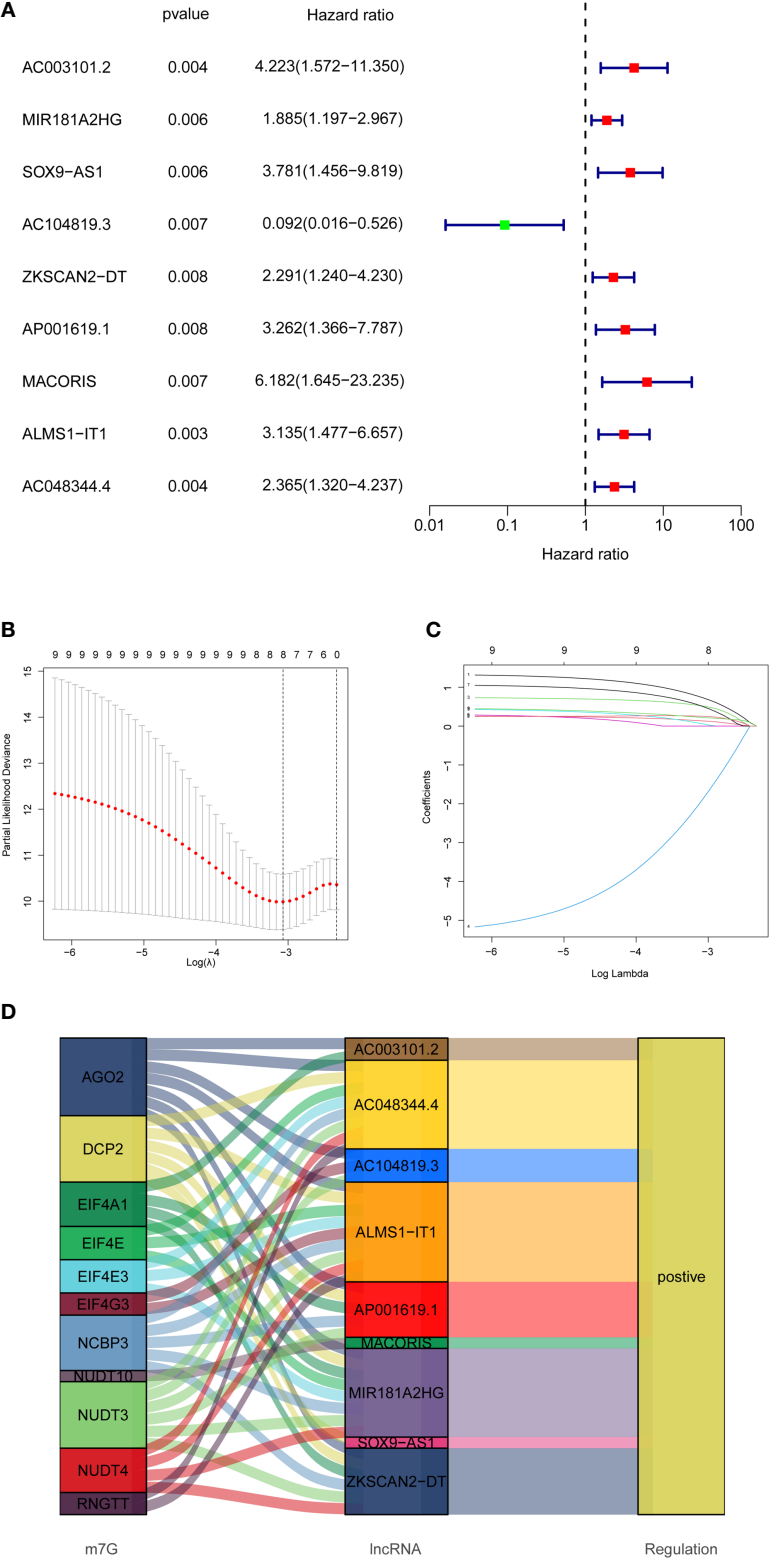
Establishment of prognostic features of m7G-related lncRNAs in COAD. **(A)** 9 prognosis-related lncRNAs screened by univariate Cox regression analysis (P<0.01). **(B)** Cross-validation for tuning the parameter selection in the LASSO regression. **(C)** LASSO coefficient distribution of 8 m7G-related lncRNAs. **(D)** Sankey diagram showing the regulatory relationship of prognosis-related lncRNAs and m7G-related genes.

**Figure 4:**
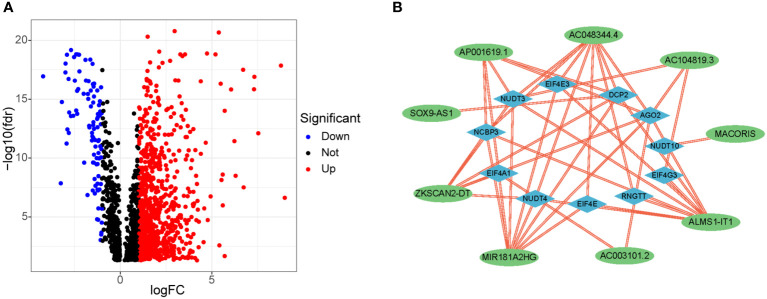
Differential expression of m7G-related lncRNAs and co-expression network of prognosis-related lncRNAs. **(A)** 903 differentially expressed m7G-related lncRNAs. **(B)** Co-expression network of prognosis -related lncRNAs and m7G-related genes.

In total, 358 colon cancer samples were randomly subdivided into a training set and validation set based on the ratio of 1:1. In the training group, the DELs expression data and survival data were integrated, and their prognostic potential was evaluated using univariate cox regression. Consequently, 9 m7G-related lncRNAs were significantly linked to prognosis (**Figure 3A**). A lncRNA gene co-expression network was established to evaluate a relationship between these 9 lncRNAs and m7G-related genes (**Figure 4B**). The Sankey diagram revealed that 9 lncRNAs were positively regulated by the corresponding genes (**Figure 3D**). The 9 prognosis-related lncRNAs were subjected to LASSO-Cox regression analysis, and 5 candidates were eventually selected to establish the optimal risk scoring model. (**Figures 3B, C**) shows the cvfit and lambda curves. Therefore, the following risk score formula was used:


risk score = AC003101.2*1.56560+AC104819.3*(−4.72897) + ZKSCAN2−DT*0.81518+MACORIS*1.33419+AC048344.4*0.87363.

**Figure 5:**
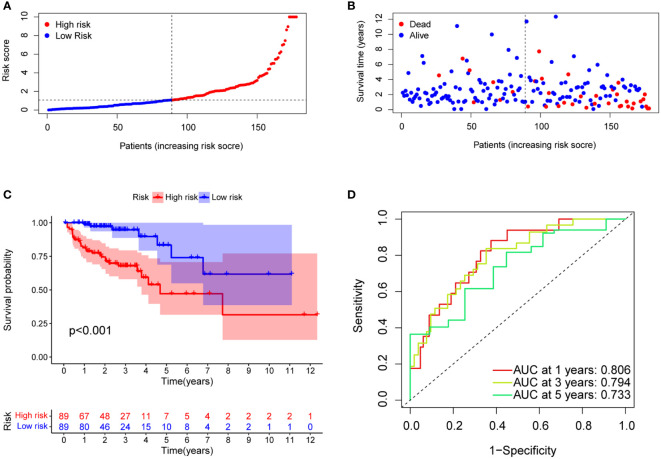
Model characteristics of m7G-related lncRNAs in the training group. **(A)** Distribution of risk scores. **(B)** Distribution of overall survival status. **(C)** Survival curves of high and low risk groups. **(D)** ROC curves for 1 year, 3 years and 5 years.

**Figure 6:**
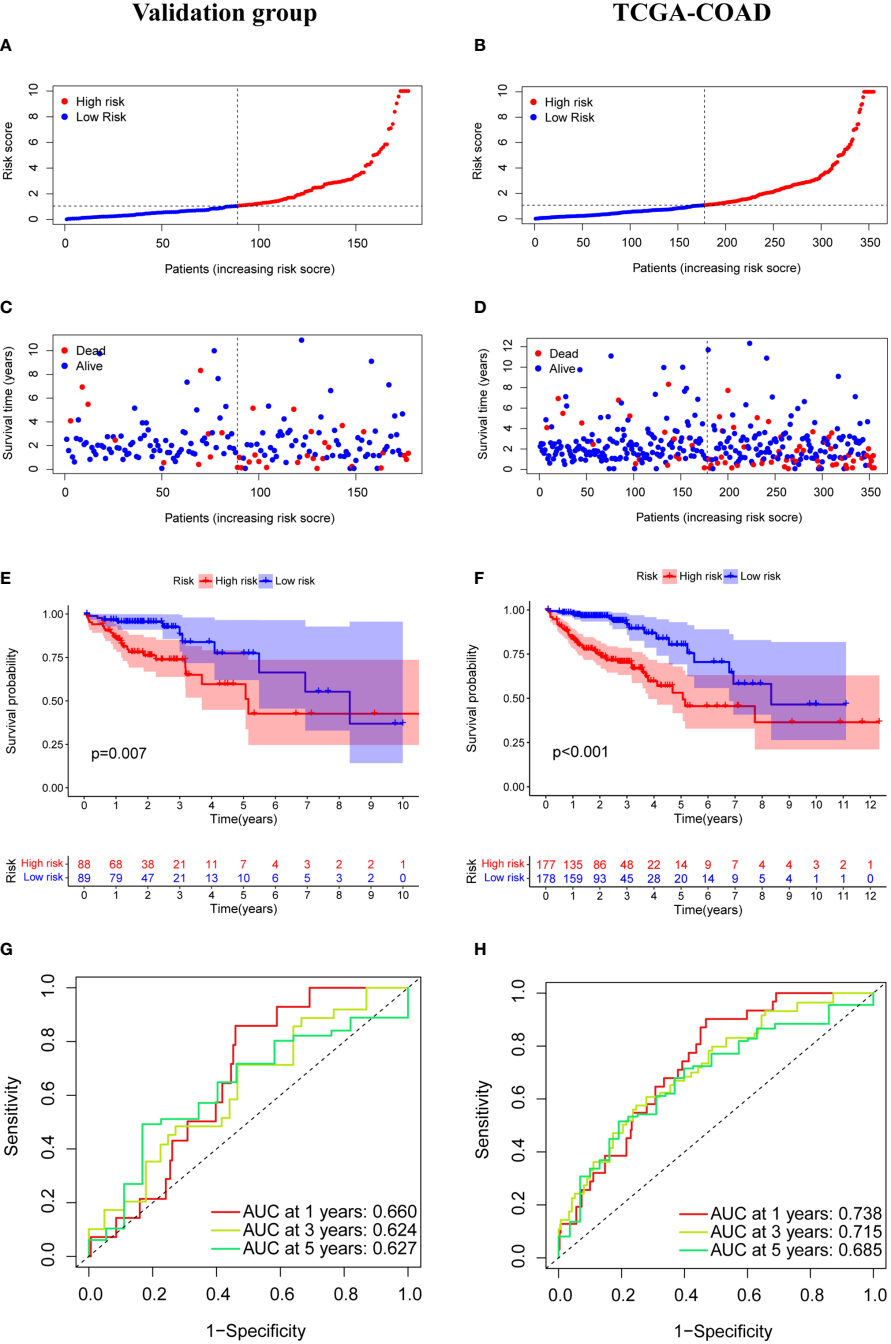
Model characteristics of m7G-related lncRNAs in the validation group and TCGA-COAD. **(A, B)** Distribution of risk scores. **(C, D)** Distribution of overall survival status. **(E, F)** Survival curves of high and low risk groups. **(G, H)** ROC curves for 1 year, 3 years and 5 years.

The median risk score was calculated based on the above formula. The TCGA-COAD cohort, training group, and validation group were divided into low-risk and high-risk subgroups; we then observed the risk score distribution and survival status distribution, respectively (**Figures 5A, B**, **6A-D**). The above results show a reasonable sample distribution of the two risk groups. Kaplan-Meier survival analysis demonstrated that the high-risk group had shorter overall survival than the low-risk group (**Figures 5C**, **6E, F**). Also, the time-dependent ROC curves revealed excellent performance in TCGA-COAD, training group, and validation group (**Figures 5D**, **6G, H**). Specifically, the training group had an area under the curve of 0.806, 0.794, and 0.733 at 1 year, 3 years, and 5 years, respectively.

### Clinical application value of the model

**Figure 7:**
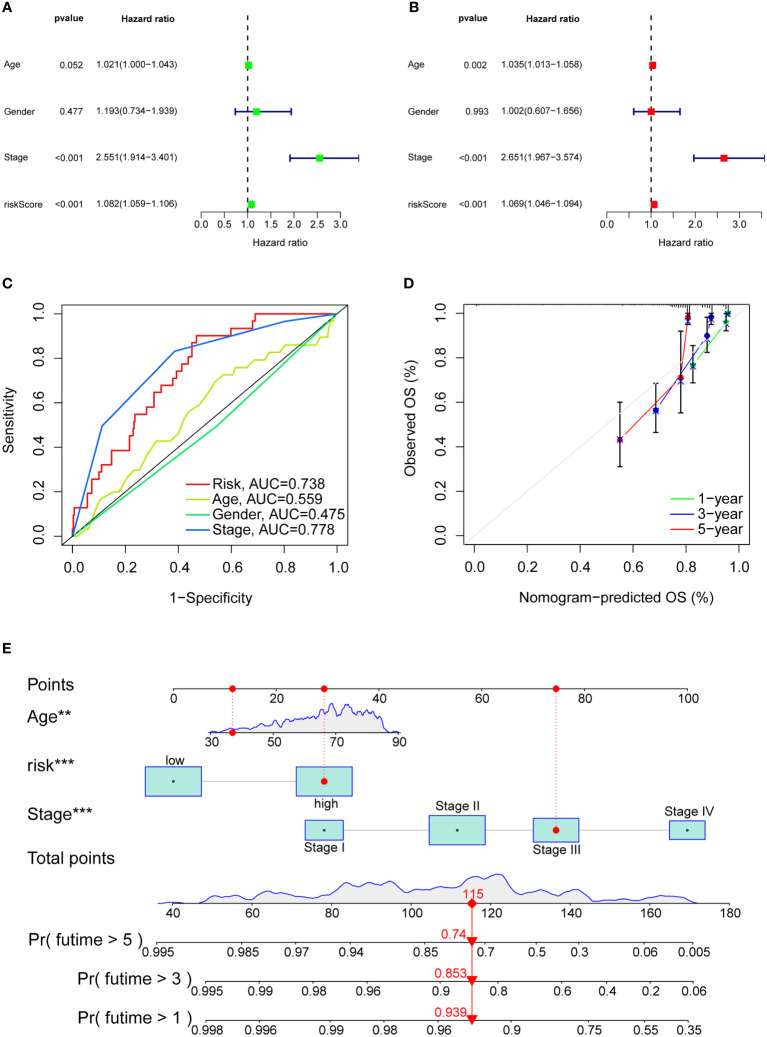
Clinical value of risk characteristics in TCGA-COAD. **(A)** Univariate Cox regression analysis of risk scores and clinical factors. **(B)** Multivariate Cox regression analysis of risk scores and clinical factors. **(C)** Clinicopathological features and the predictive accuracy of risk models. **(D)** Calibration curves test the agreement between actual and predicted results at 1, 3, and 5 years. **(E)** The nomogram that integrated the age, risk score and tumor stage predicted the probability of the 1-, 3-, and 5-year OS. **p< 0.01, ***p< 0.001.

Univariate, multivariate regression analyses on the TCGA-COAD cohort were performed to evaluate whether the model remains an independent prognostic factor after including other variables. Univariate Cox regression results (**Figure 7A**) revealed that the risk score calculated by the model is associated with the overall survival in colon adenocarcinoma patients (P<0.001). Multivariate cox regression results revealed that risk score, pathological stage, and age could be utilized as independent prognostic factors for predicting overall survival in patients with colon adenocarcinoma (**Figure 7B**). Also, we constructed ROC curves of clinicopathological features and risk scores and observed good predictive power for pathological stage and risk scores (**Figure 7C**). The nomogram combined with clinicopathological features and risk scores predicted 1-, 3-, and 5-year survival. A calibration curve was then drawn to evaluate the accuracy of the nomogram (**Figures 7D, E**).

### Molecular function and pathway discovery through GSEA

**Figure 8:**
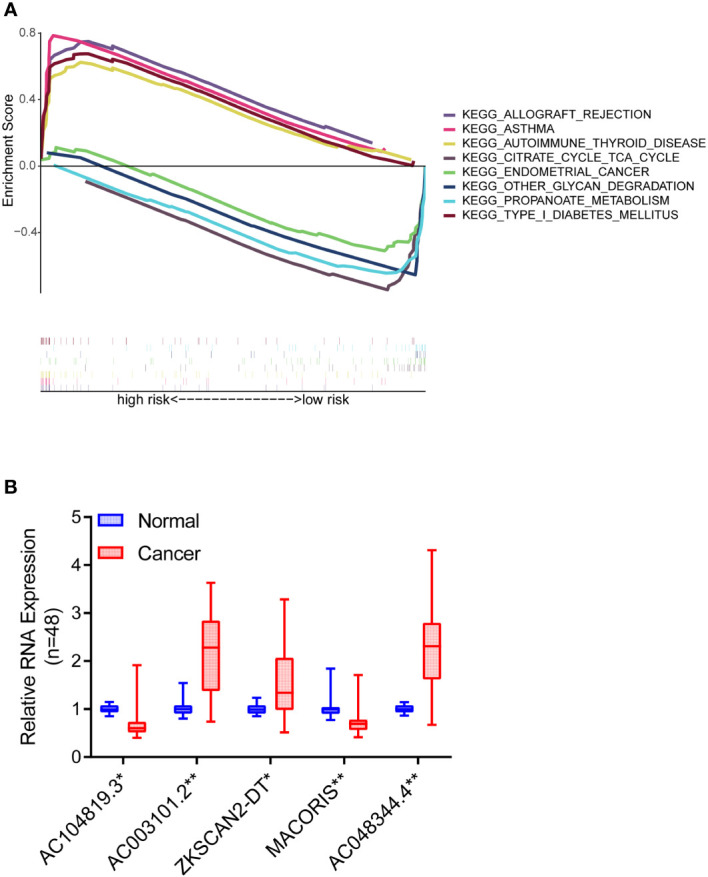
Pathway differences and model validation. **(A)** GSEA reveals pathway differences between high and low risk groups of COAD patients. **(B)** The expression differences of the five lncRNAs between 48 pairs of COAD tissues and paracancerous tissues. *P < 0.05, **P < 0.01.

GSEA software was used to explore the high-risk and low-risk groups of the KEGG pathway in the whole collection. The first four pathways enriched in the high-risk group were closely related to immune diseases, including autoimmune thyroid disease, allograft rejection, asthma, and type I diabetes (**Figure 8A**). The first four pathways enriched in the low-risk group included peroxisome, valine, leucine, isoleucine degradation, starch metabolism, sucrose metabolism and retinol metabolism. In high-risk group and low-risk group, P< 0.05 and FDR< 0.25 were considered significant enrichment.

### Extended application of risk model

**Figure 9:**
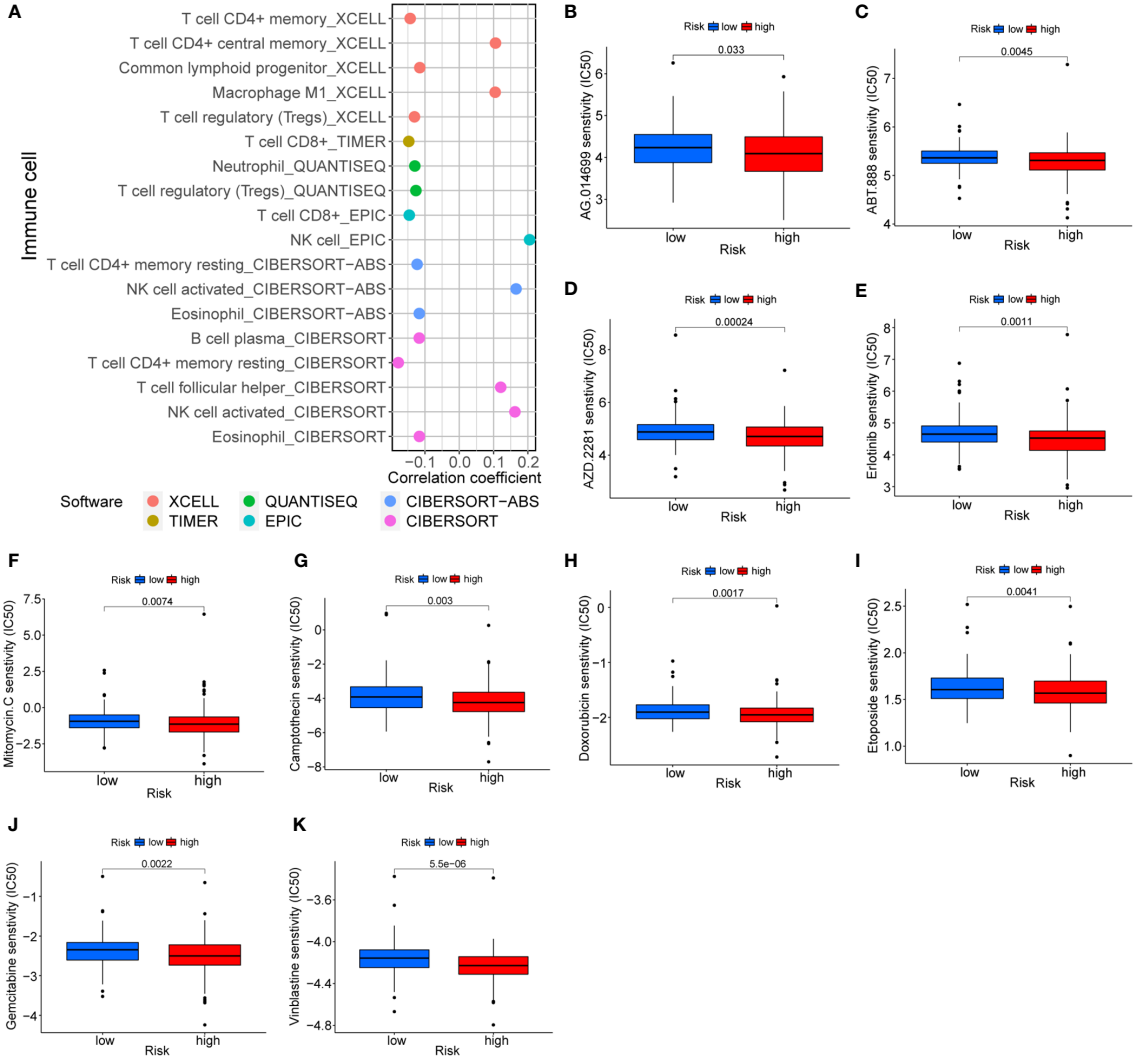
Treatment prediction based on risk models. **(A)** Correlation of immune cells and risk scores. **(B-K)** Sensitive drugs in high-risk groups.

The follow-up analysis was based on the perspective of immune cell infiltration and antitumor drugs in order to extend the clinical utility of the risk model. Risk scores were negatively correlated with most immune cells, as shown in **Figure 9A**. Consequently, low-risk individuals have a higher proportion of immune cells, in particular CD8+ T cells. There is no doubt that immunotherapy is more effective with a high level of immune cell infiltration. It is worth mentioning that there were differences in sensitive drugs in different risk groups. In comparison with the low-risk group, the high-risk group was generally more sensitive to antitumor drugs, especially PARP inhibitors (**Figures 9B-D**), EGFR inhibitors (**Figure 9E**) and chemotherapy drugs (**Figures 9F-K**), etc. A number of chemotherapy drugs have been found to be more effective in the high-risk group, including mitomycin C, camptothecin, doxorubicin, etoposide, vinblastine, and gemcitabine, with no unexpected findings in the low-risk group.

### Validation of m7G-related lncRNAs based on prognostic model

To further verify the accuracy of the above bioinformatics studies, qRT-PCR primers were designed for these five lncRNAs. Then, qRT-PCR was used to detect the expression differences of the five lncRNAs between 48 pairs of COAD tissues and paracancerous tissues. The findings demonstrated that AC003101.2, ZKSCAN2-DT and AC048344.4 levels in the tumor tissue were significantly higher than in the adjacent normal tissue. In contrast, the expression levels of AC104819.3 and MACORIS in the tumor tissue were significantly lower than those in the adjacent normal tissue (**Figure 8B**).

## Discussion

In recent years, colon adenocarcinoma classification has advanced from conventional pathological staging to molecular features and immunological research (6, 26, 27). Immunotherapy is a novel therapy developed in the past 10 years (28). Since then, it has yielded significant results in an increasing number of cancer therapies. Nonetheless, studies on tumor-immune interactions in colon adenocarcinoma are remarkably limited.

This study adopted the ConsensuClusterPlus algorithm to subdivide COAD patients into two clusters with significant differences in overall survival, tumor microenvironment scores, immune cell infiltration, and immune checkpoint expression. We introduced the concepts of cold and hot tumors. Hot tumors harbor high tumor microenvironment scores, immune cell infiltration, and immune checkpoint expression; hence they exhibit better immunotherapy effects. This confirms that hot tumors are susceptible to immunotherapy (29). Therefore, it is necessary to convert a cold tumor to a hot tumor to provide immunotherapy instead of other treatments (28). The concept of cold and hot tumors can effectively differentiate the response to immunotherapy. With the advent of immunotherapy, we are confident that this concept can effectively guide clinical treatment.

Additionally, we established a prognostic model including 5 lncRNAs, with a stronger capacity to predict prognosis than the classical TNM staging. AC003101.2, ZKSCAN2-DT, and AC048344.4 were highly expressed in tumors, whereas MACORIS and AC104819.3 were lowly expressed in tumors. Notably, AC003101.2 is involved in constructing this model; it predicts the survival rate of colorectal cancer patients and acts as a ceRNA (30). Studies on the role of AGO2 associated with AC003101.2 in tumorigenesis and tumor progression have matured. AGO2 upregulation has been discovered in several tumor types, including breast, colon adenocarcinoma, hepatocellular carcinoma, and gastric carcinoma (31–34). The Sankey diagram revealed that AC003101.2 promotes EIF4A1. EIF4A1 is implicated in epithelial-mesenchymal transition as well as metastasis of gastric and pancreatic cancers. Besides, its high expression often represents a poor prognosis (35, 36). Here, PubMed was not used in searching the related literature of the other 4 lncRNAs (AC104819.3, ZKSCAN2-DT, MACORIS, AC048344.4). Therefore, we propose that these 4 lncRNAs are linked to poor prognosis in colon adenocarcinoma for the first time.

Gene Set Enrichment Analysis (GSEA) was performed on the two risk groups to explore the biological functions of different risk subgroups. Consequently, the low-risk group had enrichment of metabolic-related pathways. In contrast, the high-risk group had enrichment in several immune-related diseases, including asthma, autoimmune thyroid disease, allograft rejection, and type I diabetes. Therefore, we speculate that patients with colon adenocarcinoma co-existing with the immune-related disease may represent a poor prognosis.

To confirm the accuracy of the prognostic model based on five lncRNAs, the expression levels of these five lncRNAs in tumor tissues and adjacent tissues were detected using qRT-PCR. Consequently, five lncRNAs were differentially expressed in normal tissues and tumor tissues. Besides, the expression levels of AC104819.3 and MACORIS in tumor tissues were significantly lower than those in adjacent normal tissues. In contrast, AC003101.2, ZKSCAN2-DT and C048344.4 in tumor tissues were significantly higher than in adjacent normal tissues. Despite the PCR detection on 48 pairs of clinical samples, the sample size was limited. Moreover, the underlying mechanism by which lncRNAs influence m7G remains unknown and warrants additional studies.

## Conclusion

In conclusion, we constructed a robust prognostic prediction model comprising only five lncRNAs, and more clinically applicable than other classical clinical features. For the first time, we found that AC003101.2, AC104819.3, ZKSCAN2-DT, MACORIS, and AC048344.4 can be used as biomarkers to predict the prognosis patients with colon adenocarcinoma. This will help in the clinical diagnosis and treatment of colon adenocarcinoma.

## Data availability statement

The original contributions presented in the study are included in the article/**Supplementary Material**. Further inquiries can be directed to the corresponding authors.

## Ethics statement

This study was reviewed and approved by Ethics Committee of Affiliated Hospital of Xuzhou Medical University. The patients/participants provided their written informed consent to participate in this study.

## Author contributions

ZC and JW made the same contribution to this research. ZC and JW contributed to conceptualization. ZC contributed to methodology and manuscript preparation. JW contributed to the experimental section. YX and TJ contributed to data analysis. ZX, SL, and YZ contributed to visualization. ZC, JW, JS and HS reviewed and edited the manuscript. JS contributed to project management and funding access. All authors contributed to the article and approved the submitted version.

## Funding

This study was supported by National Natural Science Foundation of China (82073133) and Natural Science Foundation Project of Jiangsu Province (BK20191154).

## Acknowledgments

We sincerely acknowledge The Cancer Genome Atlas (TCGA) for providing transcriptomic and clinicopathological data.

## Conflict of interest

The authors declare that the research was conducted in the absence of any commercial or financial relationships that could be construed as a potential conflict of interest.

## Publisher’s note

All claims expressed in this article are solely those of the authors and do not necessarily represent those of their affiliated organizations, or those of the publisher, the editors and the reviewers. Any product that may be evaluated in this article, or claim that may be made by its manufacturer, is not guaranteed or endorsed by the publisher.

## Supplementary material

Supplementary Figure 1 Differences in clinicopathological factors and immune cell infiltration among different clusters. (A)The heatmap shows the distribution of pathological stage, gender, and age in different clusters, and there are obvious differences in pathological stage. (B) Heatmap showing the distribution of immune cells in different clusters in different sites.

Supplementary Table 1 Forty m7G methylation-related genes.

Supplementary Table 2 Primer sequences of the 5 lncRNAs used to validate the model.

Supplementary Table 3 P-values for immune cells in different clusters.
