# Supplementary figures and images for: N7-methylguanosine-related lncRNAs: Distinction between hot and cold tumors and construction of predictive models in colon adenocarcinoma
Source: Front Oncol. 2022 Sep 15;12:951452. doi: 10.3389/fonc.2022.951452 (PMC9520617; doi:10.3389/fonc.2022.951452)

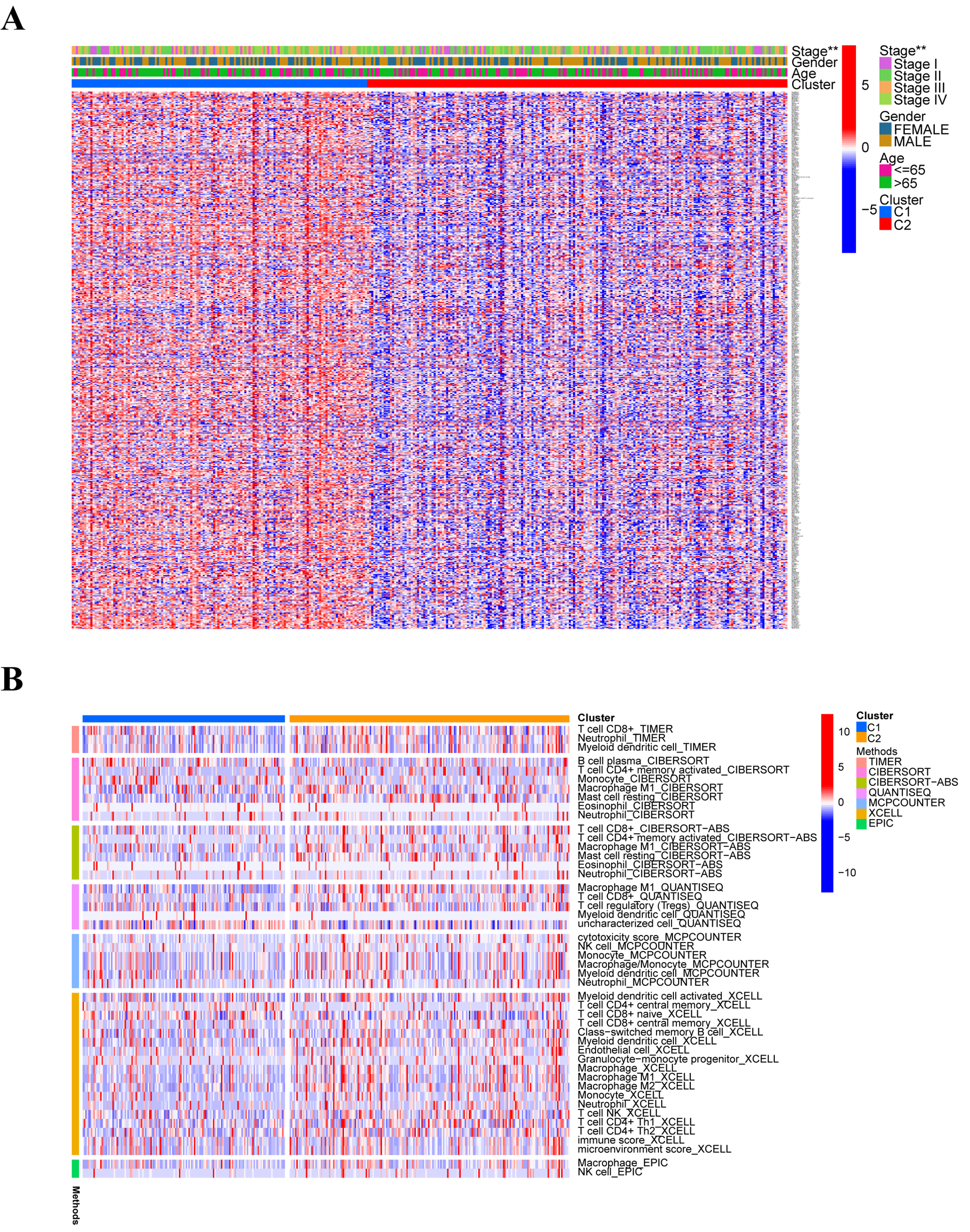

Supplement: Supplementary Figure 1 — Differences in clinicopathological factors and immune cell infiltration among different clusters. (A)The heatmap shows the distribution of pathological stage, gender, and age in different clusters, and there are obvious differences in pathological stage. (B) Heatmap showing the distribution of immune cells in different clusters in different sites. [file Image_1.tif]
